# Crystal structure of (*E*)-5-benz­yloxy-2-{[(4-nitro­phen­yl)imino]­meth­yl}phenol

**DOI:** 10.1107/S2056989015022173

**Published:** 2015-11-28

**Authors:** Nadir Ghichi, Mohamed Amine Benaouida, Ali Benboudiaf, Hocine Merazig

**Affiliations:** a Unité de Recherche de Chimie de l’Environnement et Moléculaire Structurale (CHEMS), Faculté des Sciences Exactes, Département de Chimie, Université Constantine 1, Algeria

**Keywords:** crystal structure, enol, imine, Schiff base, hydrogen bonding

## Abstract

In the title compound, C_20_H_16_N_2_O_4_, the mol­ecule adopts an *E* conformation about the N=C bond. There is an intra­molecular O—H⋯N hydrogen bond forming an *S*(6) ring motif. The nitro­benzene and benz­yloxy rings are inclined to the central benzene ring by 4.34 (10) and 27.66 (11)°, respectively, and to one another by 31.40 (12)°. In the crystal, mol­ecules are linked *via* C—H⋯O hydrogen bonds, forming zigzag chains along [001]. Within the chains there are C—H⋯π inter­actions present. The chains are linked *via* π–π inter­actions [inter-centroid distance = 3.7048 (15) Å], forming slabs parallel to the *bc* plane.

## Related literature   

For the use of Schiff bases in synthesis, see: Arora *et al.* (2002 ▸). For thermochromic, photochromic, biological and pharmacological activities of Schiff base compounds and their derivatives, see: Khandar *et al.* (2005 ▸); Tarafder *et al.* (2002 ▸); Hadjoudis *et al.* (1987 ▸). Schiff bases have been reported to show anti­cancer activity (Desai *et al.*, 2001 ▸). For a related structure, see: Tzimopoulos *et al.* (2010 ▸). 
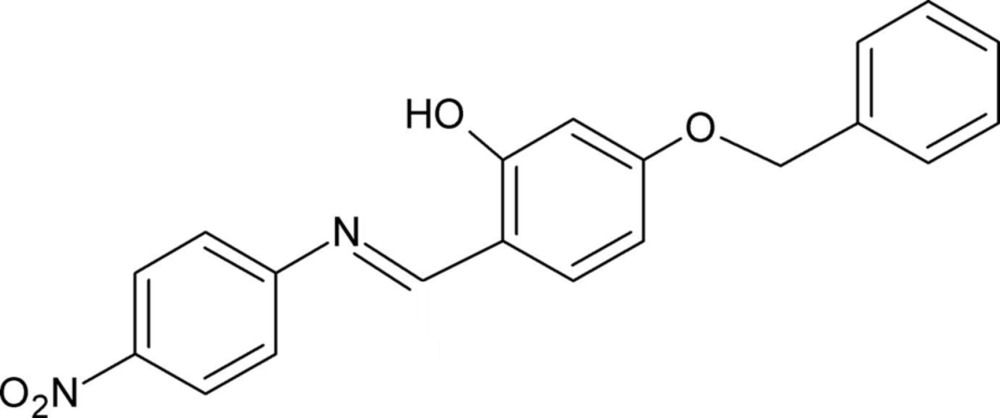


## Experimental   

### Crystal data  

C_20_H_16_N_2_O_4_
*M* *_r_* = 348.35
Monoclinic, 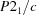

*a* = 15.3407 (5) Å
*b* = 9.5618 (3) Å
*c* = 11.7616 (4) Å
β = 100.615 (1)°
*V* = 1695.72 (10) Å^3^
*Z* = 4
Mo *K*α radiation
μ = 0.10 mm^−1^
*T* = 293 K
0.03 × 0.02 × 0.01 mm

### Data collection  

Bruker APEXII CCD diffractometer
14260 measured reflections
3302 independent reflections
2389 reflections with *I* > 2σ(*I*)
*R*_int_ = 0.020

### Refinement  

*R*[*F* ^2^ > 2σ(*F* ^2^)] = 0.049
*wR*(*F* ^2^) = 0.146
*S* = 1.04
3302 reflections
239 parameters
H atoms treated by a mixture of independent and constrained refinement
Δρ_max_ = 0.19 e Å^−3^
Δρ_min_ = −0.13 e Å^−3^

### 

Data collection: *APEX2* (Bruker, 2006 ▸); cell refinement: *SAINT* (Bruker, 2006 ▸); data reduction: *SAINT*; program(s) used to solve structure: *SHELXS97* (Sheldrick, 2008 ▸); program(s) used to refine structure: *SHELXL2014* (Sheldrick, 2015 ▸); molecular graphics: *ORTEP-3 for Windows* (Farrugia, 2012 ▸); software used to prepare material for publication: *WinGX* (Farrugia, 2012 ▸).

## Supplementary Material

Crystal structure: contains datablock(s) I, global. DOI: 10.1107/S2056989015022173/su5239sup1.cif

Structure factors: contains datablock(s) I. DOI: 10.1107/S2056989015022173/su5239Isup2.hkl

Supporting information file. DOI: 10.1107/S2056989015022173/su5239Isup3.cml

**Fig. 1.**
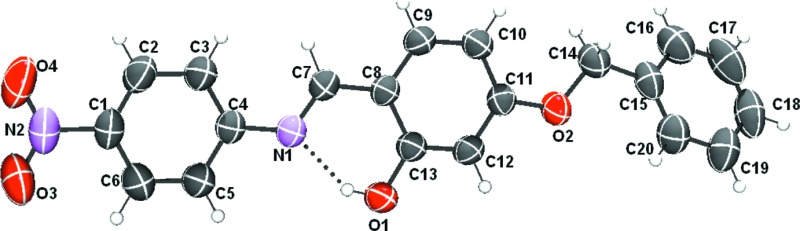
View of the molecular structure of the title compound, with atom labelling. Displacement ellipsoids are drawn at the 50% probability level. The intramolecular hydrogen bond is shown as a dashed line (see Table 1).

**Fig. 2.**
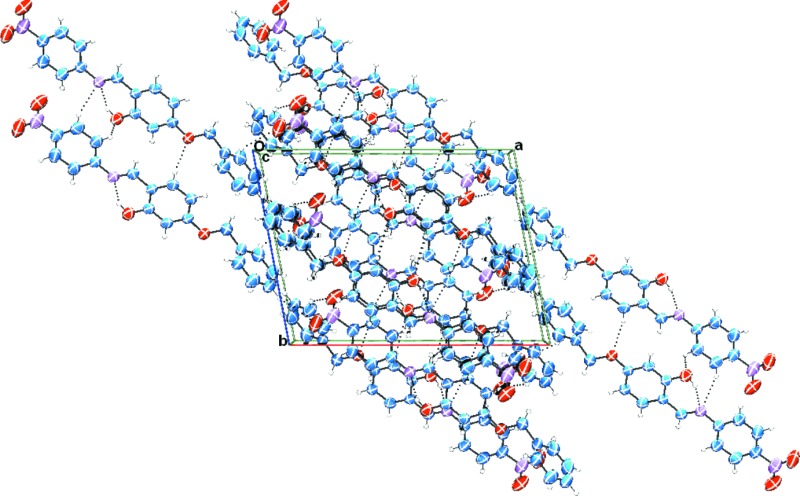
A view along the c axis of the crystal packing of the title compound, showing the hydrogen bonds as dashed lines (see Table 1).

. DOI: 10.1107/S2056989015022173/su5239fig1.tif
View of the mol­ecular structure of the title compound, with atom labelling. Displacement ellipsoids are drawn at the 50% probability level. The intra­molecular hydrogen bond is shown as a dashed line (see Table 1).

c . DOI: 10.1107/S2056989015022173/su5239fig2.tif
A view along the *c* axis of the crystal packing of the title compound, showing the hydrogen bonds as dashed lines (see Table 1).

CCDC reference: 1437973

Additional supporting information:  crystallographic information; 3D view; checkCIF report

## Figures and Tables

**Table 1:** Hydrogen-bond geometry (Å, °) *Cg*3 is the centroid of the C15–C20 ring.

*D*—H⋯*A*	*D*—H	H⋯*A*	*D*⋯*A*	*D*—H⋯*A*
O1—H1⋯N1	0.82	1.87	2.599 (2)	148
C9—H9⋯O2^i^	0.93	2.56	3.476 (2)	168
C10—H10⋯*Cg*3^i^	0.93	2.87	3.754 (2)	159

Symmetry code: (i) 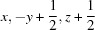.

## supplementary crystallographic information

### S1. Commentary

Schiff bases are extremely useful in the preparation of various compounds (Arora *et al.*, 2002), and have been shown to have photochromic and thermochromic properties (Hadjoudis *et al.*, 1987). The importance of imine derivatives has increased due to the fact that they have been shown to have anti-cancer properties (Desai *et al.*, 2001; Khandar *et al.*, 2005). The presence of a nitro group in various molecules and Schiff base derivatives, especially in the *p*-position, has an influence on the effectiveness of bacteriostatics (Tarafder *et al.*, 2002). The title Schiff base incorporates a nitro group in the *p*-position and herein we report on its synthesis and crystal structure.

The molecular structure of the title compound is show in Fig 1. The molecule adopts an *E* conformation about the N1═C7 bond [1.284 (2) Å]. The nitro­benzene and benzyl­oxy rings are inclined to the central benzene ring by 4.34 (10) and 27.66 (11) °, respectively, and to one another by 31.40 (12) °. There is an intra­molecular O—H···N hydrogen bond forming an S(6) ring motif (Table 1).

In the crystal, molecules are linked via N—H···O hydrogen bonds forming zigzag chains along [001]. Within the chains there are C—H···π inter­actions present (Table 1 and Fig. 2). The chains are linked via slipped parallel π–π inter­actions forming slabs parallel to the *bc* plane [Cg3···Cg3^i^ = 3.7048 (15) Å; Cg3 is the centroid of ring C15—C20; inter-planar distance = 3.600 (11) Å; slippage = 0.572 Å; symmetry code: (i) -x + 2, -y + 1, -z].

### S2. Synthesis and crystallisation

A mixture of 4-nitro­benzene­amine and 4-benzyl­oxy-2-hy­droxy­benzaldehyde in ethanol or methanol was refluxed for 2 h. On completion of the reaction, the orange precipitate formed was crystallized in a mixture of tetra­hydro­furan and chloro­form (1:2), giving very small orange crystals after one week.

### S3. Refinement

Crystal data, data collection and structure refinement details are summarized in Table 2. Methine H atom H7 was freely refined. The OH and other C-bound H atoms were included in calculated positions and treated as riding atoms: O—H = 0.82 Å, C—H = 0.93-1.00 Å with *U*_iso_(H) = 1.5*U*_eq_(O) and 1.2*U*_eq_(C) for other H atoms.

### Crystal data

**Table:**

C_20_H_16_N_2_O_4_	*Z* = 4
*M**_r_* = 348.35	*F*(000) = 728
Monoclinic, *P*2_1_/*c*	*D*_x_ = 1.364 Mg m^−^^3^
*a* = 15.3407 (5) Å	Mo *K*α radiation, λ = 0.71073 Å
*b* = 9.5618 (3) Å	µ = 0.10 mm^−^^1^
*c* = 11.7616 (4) Å	*T* = 293 K
β = 100.615 (1)°	Block, orange
*V* = 1695.72 (10) Å^3^	0.03 × 0.02 × 0.01 mm

### Data collection

**Table:**

Bruker APEXII CCD diffractometer	2389 reflections with *I* > 2σ(*I*)
Radiation source: sealed tube	*R*_int_ = 0.020
Graphite monochromator	θ_max_ = 26.0°, θ_min_ = 2.8°
phi and ω scans	*h* = −18→18
14260 measured reflections	*k* = −10→11
3302 independent reflections	*l* = −11→14

### Refinement

**Table:**

Refinement on *F*^2^	Primary atom site location: structure-invariant direct methods
Least-squares matrix: full	Secondary atom site location: difference Fourier map
*R*[*F*^2^ > 2σ(*F*^2^)] = 0.049	Hydrogen site location: mixed
*wR*(*F*^2^) = 0.146	H atoms treated by a mixture of independent and constrained refinement
*S* = 1.04	*w* = 1/[σ^2^(*F*_o_^2^) + (0.0624*P*)^2^ + 0.5493*P*] where *P* = (*F*_o_^2^ + 2*F*_c_^2^)/3
3302 reflections	(Δ/σ)_max_ < 0.001
239 parameters	Δρ_max_ = 0.19 e Å^−^^3^
0 restraints	Δρ_min_ = −0.13 e Å^−^^3^

### Special details

**Table:**

Geometry. Bond distances, angles *etc*. have been calculated using the rounded fractional coordinates. All su's are estimated from the variances of the (full) variance-covariance matrix. The cell e.s.d.'s are taken into account in the estimation of distances, angles and torsion angles
Refinement. Refinement on *F*^2^ for ALL reflections except those flagged by the user for potential systematic errors. Weighted *R*-factors *wR* and all goodnesses of fit *S* are based on *F*^2^, conventional *R*-factors *R* are based on *F*, with *F* set to zero for negative *F*^2^. The observed criterion of *F*^2^ > σ(*F*^2^) is used only for calculating -*R*-factor-obs *etc*. and is not relevant to the choice of reflections for refinement. *R*-factors based on *F*^2^ are statistically about twice as large as those based on *F*, and *R*-factors based on ALL data will be even larger.

### Fractional atomic coordinates and isotropic or equivalent isotropic displacement parameters (Å^2^)

**Table:**

	*x*	*y*	*z*	*U*_iso_*/*U*_eq_	
O1	0.47868 (9)	0.50409 (16)	0.17069 (12)	0.0717 (5)	
O2	0.75000 (8)	0.39891 (15)	0.06771 (11)	0.0637 (5)	
O3	0.11551 (13)	0.4282 (3)	0.61135 (19)	0.1135 (9)	
O4	0.20529 (14)	0.3185 (2)	0.74236 (18)	0.1087 (9)	
N1	0.44170 (10)	0.40083 (16)	0.36061 (13)	0.0544 (5)	
N2	0.18708 (14)	0.3734 (2)	0.6477 (2)	0.0786 (8)	
C1	0.25402 (13)	0.3766 (2)	0.57425 (18)	0.0618 (7)	
C2	0.33415 (15)	0.3141 (2)	0.61163 (18)	0.0701 (8)	
C3	0.39794 (14)	0.3190 (2)	0.54255 (17)	0.0676 (7)	
C4	0.38141 (12)	0.38758 (18)	0.43716 (15)	0.0528 (6)	
C5	0.29945 (14)	0.4494 (3)	0.40209 (19)	0.0735 (8)	
C6	0.23565 (14)	0.4436 (3)	0.4701 (2)	0.0792 (9)	
C7	0.51738 (12)	0.33921 (19)	0.37785 (16)	0.0525 (6)	
C8	0.57800 (11)	0.35446 (18)	0.29908 (14)	0.0483 (5)	
C9	0.66038 (12)	0.2898 (2)	0.32123 (15)	0.0568 (6)	
C10	0.72109 (12)	0.3028 (2)	0.24852 (15)	0.0567 (6)	
C11	0.69764 (11)	0.38241 (19)	0.14824 (14)	0.0500 (5)	
C12	0.61596 (12)	0.4477 (2)	0.12285 (15)	0.0543 (6)	
C13	0.55691 (11)	0.43658 (19)	0.19724 (14)	0.0508 (5)	
C14	0.83798 (13)	0.3485 (3)	0.09251 (18)	0.0690 (7)	
C15	0.88052 (12)	0.3718 (2)	−0.01124 (17)	0.0624 (7)	
C16	0.93985 (15)	0.2759 (3)	−0.0378 (2)	0.0834 (9)	
C17	0.98237 (16)	0.2974 (4)	−0.1306 (3)	0.0970 (13)	
C18	0.96510 (16)	0.4138 (4)	−0.1971 (2)	0.0912 (10)	
C19	0.90594 (17)	0.5082 (3)	−0.1720 (2)	0.0876 (10)	
C20	0.86397 (15)	0.4885 (3)	−0.0794 (2)	0.0762 (8)	
H1	0.44953	0.48980	0.22138	0.1076*	
H2	0.34574	0.26878	0.68277	0.0842*	
H3	0.45245	0.27576	0.56716	0.0811*	
H5	0.28726	0.49561	0.33137	0.0882*	
H6	0.18052	0.48488	0.44544	0.0950*	
H7	0.5357 (12)	0.276 (2)	0.4452 (17)	0.062 (5)*	
H9	0.67529	0.23560	0.38762	0.0681*	
H10	0.77623	0.25960	0.26603	0.0680*	
H12	0.60088	0.49934	0.05509	0.0652*	
H14A	0.83806	0.24960	0.11074	0.0829*	
H14B	0.87111	0.39755	0.15887	0.0829*	
H16	0.95179	0.19552	0.00689	0.1000*	
H17	1.02292	0.23183	−0.14745	0.1165*	
H18	0.99370	0.42830	−0.25926	0.1093*	
H19	0.89342	0.58753	−0.21781	0.1051*	
H20	0.82390	0.55508	−0.06297	0.0914*	

### Atomic displacement parameters (Å^2^)

**Table:**

	*U*^11^	*U*^22^	*U*^33^	*U*^12^	*U*^13^	*U*^23^
O1	0.0639 (8)	0.0925 (11)	0.0626 (8)	0.0249 (7)	0.0218 (7)	0.0220 (7)
O2	0.0551 (7)	0.0892 (10)	0.0499 (7)	0.0068 (6)	0.0181 (6)	0.0035 (6)
O3	0.0819 (12)	0.1413 (18)	0.1321 (17)	0.0066 (12)	0.0585 (12)	0.0054 (14)
O4	0.1263 (16)	0.1218 (16)	0.0952 (13)	−0.0091 (12)	0.0656 (12)	0.0136 (12)
N1	0.0550 (8)	0.0608 (9)	0.0494 (8)	0.0015 (7)	0.0149 (7)	0.0025 (7)
N2	0.0811 (13)	0.0727 (12)	0.0937 (15)	−0.0168 (10)	0.0470 (12)	−0.0127 (11)
C1	0.0665 (12)	0.0587 (11)	0.0671 (12)	−0.0115 (9)	0.0302 (10)	−0.0083 (9)
C2	0.0811 (14)	0.0734 (13)	0.0615 (12)	0.0013 (11)	0.0279 (11)	0.0114 (10)
C3	0.0662 (12)	0.0766 (14)	0.0640 (12)	0.0102 (10)	0.0226 (10)	0.0130 (10)
C4	0.0555 (10)	0.0540 (10)	0.0512 (10)	−0.0028 (8)	0.0158 (8)	−0.0027 (8)
C5	0.0669 (12)	0.0925 (16)	0.0651 (13)	0.0143 (11)	0.0225 (10)	0.0187 (11)
C6	0.0610 (12)	0.0987 (17)	0.0827 (15)	0.0133 (11)	0.0258 (11)	0.0124 (13)
C7	0.0574 (10)	0.0537 (10)	0.0476 (10)	−0.0012 (8)	0.0132 (8)	0.0031 (8)
C8	0.0526 (9)	0.0518 (9)	0.0416 (9)	−0.0008 (7)	0.0117 (7)	−0.0008 (7)
C9	0.0600 (10)	0.0633 (11)	0.0470 (9)	0.0056 (8)	0.0098 (8)	0.0098 (8)
C10	0.0519 (9)	0.0671 (12)	0.0514 (10)	0.0083 (8)	0.0106 (8)	0.0041 (8)
C11	0.0505 (9)	0.0587 (10)	0.0419 (9)	−0.0026 (7)	0.0118 (7)	−0.0051 (7)
C12	0.0589 (10)	0.0625 (11)	0.0423 (9)	0.0056 (8)	0.0112 (8)	0.0074 (8)
C13	0.0513 (9)	0.0547 (10)	0.0468 (9)	0.0047 (7)	0.0103 (7)	0.0018 (8)
C14	0.0561 (11)	0.0931 (15)	0.0595 (12)	0.0055 (10)	0.0148 (9)	0.0024 (10)
C15	0.0484 (10)	0.0841 (14)	0.0569 (11)	−0.0077 (9)	0.0152 (8)	−0.0163 (10)
C16	0.0680 (13)	0.1007 (18)	0.0825 (15)	0.0113 (12)	0.0169 (12)	−0.0093 (13)
C17	0.0651 (14)	0.133 (3)	0.0981 (19)	0.0109 (15)	0.0285 (14)	−0.0418 (19)
C18	0.0712 (14)	0.140 (2)	0.0700 (15)	−0.0268 (16)	0.0326 (12)	−0.0273 (16)
C19	0.0902 (16)	0.1022 (19)	0.0783 (16)	−0.0157 (14)	0.0366 (13)	−0.0003 (14)
C20	0.0745 (13)	0.0841 (16)	0.0766 (14)	0.0000 (11)	0.0316 (11)	−0.0042 (12)

### Geometric parameters (Å, º)

**Table:**

O1—C13	1.348 (2)	C14—C15	1.503 (3)
O2—C11	1.359 (2)	C15—C20	1.370 (3)
O2—C14	1.412 (2)	C15—C16	1.368 (3)
O3—N2	1.221 (3)	C16—C17	1.386 (4)
O4—N2	1.216 (3)	C17—C18	1.358 (5)
N1—C4	1.410 (2)	C18—C19	1.351 (4)
N1—C7	1.284 (2)	C19—C20	1.376 (3)
O1—H1	0.8200	C2—H2	0.9300
N2—C1	1.459 (3)	C3—H3	0.9300
C1—C2	1.365 (3)	C5—H5	0.9300
C1—C6	1.365 (3)	C6—H6	0.9300
C2—C3	1.383 (3)	C7—H7	1.00 (2)
C3—C4	1.384 (3)	C9—H9	0.9300
C4—C5	1.382 (3)	C10—H10	0.9300
C5—C6	1.375 (3)	C12—H12	0.9300
C7—C8	1.436 (3)	C14—H14A	0.9700
C8—C13	1.419 (2)	C14—H14B	0.9700
C8—C9	1.388 (3)	C16—H16	0.9300
C9—C10	1.381 (3)	C17—H17	0.9300
C10—C11	1.394 (2)	C18—H18	0.9300
C11—C12	1.382 (3)	C19—H19	0.9300
C12—C13	1.375 (2)	C20—H20	0.9300
			
C11—O2—C14	118.78 (15)	C17—C18—C19	119.3 (2)
C4—N1—C7	122.55 (16)	C18—C19—C20	120.8 (3)
C13—O1—H1	109.00	C15—C20—C19	120.7 (2)
O3—N2—O4	123.1 (2)	C1—C2—H2	120.00
O4—N2—C1	118.9 (2)	C3—C2—H2	120.00
O3—N2—C1	118.0 (2)	C2—C3—H3	120.00
N2—C1—C2	119.38 (19)	C4—C3—H3	120.00
C2—C1—C6	121.3 (2)	C4—C5—H5	119.00
N2—C1—C6	119.32 (19)	C6—C5—H5	119.00
C1—C2—C3	119.26 (19)	C1—C6—H6	120.00
C2—C3—C4	120.63 (19)	C5—C6—H6	120.00
N1—C4—C3	125.52 (17)	N1—C7—H7	121.3 (11)
N1—C4—C5	116.02 (17)	C8—C7—H7	117.0 (11)
C3—C4—C5	118.47 (18)	C8—C9—H9	119.00
C4—C5—C6	121.0 (2)	C10—C9—H9	119.00
C1—C6—C5	119.3 (2)	C9—C10—H10	121.00
N1—C7—C8	121.71 (17)	C11—C10—H10	121.00
C7—C8—C13	121.79 (16)	C11—C12—H12	120.00
C9—C8—C13	117.59 (15)	C13—C12—H12	120.00
C7—C8—C9	120.61 (16)	O2—C14—H14A	110.00
C8—C9—C10	122.57 (17)	O2—C14—H14B	110.00
C9—C10—C11	118.34 (17)	C15—C14—H14A	110.00
O2—C11—C10	124.01 (16)	C15—C14—H14B	110.00
C10—C11—C12	120.78 (16)	H14A—C14—H14B	108.00
O2—C11—C12	115.18 (15)	C15—C16—H16	120.00
C11—C12—C13	120.33 (16)	C17—C16—H16	120.00
O1—C13—C8	121.03 (15)	C16—C17—H17	120.00
O1—C13—C12	118.59 (16)	C18—C17—H17	120.00
C8—C13—C12	120.37 (16)	C17—C18—H18	120.00
O2—C14—C15	108.86 (17)	C19—C18—H18	120.00
C14—C15—C16	119.5 (2)	C18—C19—H19	120.00
C16—C15—C20	118.1 (2)	C20—C19—H19	120.00
C14—C15—C20	122.3 (2)	C15—C20—H20	120.00
C15—C16—C17	120.7 (3)	C19—C20—H20	120.00
C16—C17—C18	120.3 (3)		
			
C14—O2—C11—C10	−8.6 (3)	C13—C8—C9—C10	0.0 (3)
C14—O2—C11—C12	173.28 (18)	C7—C8—C13—O1	0.2 (3)
C11—O2—C14—C15	177.00 (17)	C7—C8—C13—C12	−179.20 (17)
C7—N1—C4—C3	5.8 (3)	C9—C8—C13—O1	−179.10 (16)
C7—N1—C4—C5	−174.31 (19)	C9—C8—C13—C12	1.5 (3)
C4—N1—C7—C8	−179.76 (16)	C8—C9—C10—C11	−1.0 (3)
O3—N2—C1—C2	−178.9 (2)	C9—C10—C11—O2	−177.44 (17)
O3—N2—C1—C6	2.0 (3)	C9—C10—C11—C12	0.6 (3)
O4—N2—C1—C2	2.2 (3)	O2—C11—C12—C13	179.08 (16)
O4—N2—C1—C6	−177.0 (2)	C10—C11—C12—C13	0.9 (3)
N2—C1—C2—C3	−179.09 (18)	C11—C12—C13—O1	178.65 (17)
C6—C1—C2—C3	0.1 (3)	C11—C12—C13—C8	−2.0 (3)
N2—C1—C6—C5	178.5 (2)	O2—C14—C15—C16	−144.8 (2)
C2—C1—C6—C5	−0.7 (4)	O2—C14—C15—C20	37.0 (3)
C1—C2—C3—C4	0.7 (3)	C14—C15—C16—C17	−177.7 (2)
C2—C3—C4—N1	179.10 (18)	C20—C15—C16—C17	0.5 (4)
C2—C3—C4—C5	−0.8 (3)	C14—C15—C20—C19	178.3 (2)
N1—C4—C5—C6	−179.7 (2)	C16—C15—C20—C19	0.1 (3)
C3—C4—C5—C6	0.2 (3)	C15—C16—C17—C18	−0.5 (4)
C4—C5—C6—C1	0.5 (4)	C16—C17—C18—C19	−0.1 (4)
N1—C7—C8—C9	178.26 (17)	C17—C18—C19—C20	0.8 (4)
N1—C7—C8—C13	−1.0 (3)	C18—C19—C20—C15	−0.7 (4)
C7—C8—C9—C10	−179.31 (17)		

### Hydrogen-bond geometry (Å, º)

Cg3 is the centroid of the C15–C20 ring.

**Table:**

*D*—H···*A*	*D*—H	H···*A*	*D*···*A*	*D*—H···*A*
O1—H1···N1	0.82	1.87	2.599 (2)	148
C9—H9···O2^i^	0.93	2.56	3.476 (2)	168
C10—H10···*Cg*3^i^	0.93	2.87	3.754 (2)	159

Symmetry code: (i) *x*, −*y*+1/2, *z*+1/2.
