# Supplementary figures and images for: Crystal structure of (E)-5-benz­yloxy-2-{[(4-nitro­phen­yl)imino]­meth­yl}phenol
Source: Acta Crystallogr E Crystallogr Commun. 2015 Nov 28;71(Pt 12):o1000–1. doi: 10.1107/S2056989015022173 (PMC4719944; doi:10.1107/S2056989015022173)

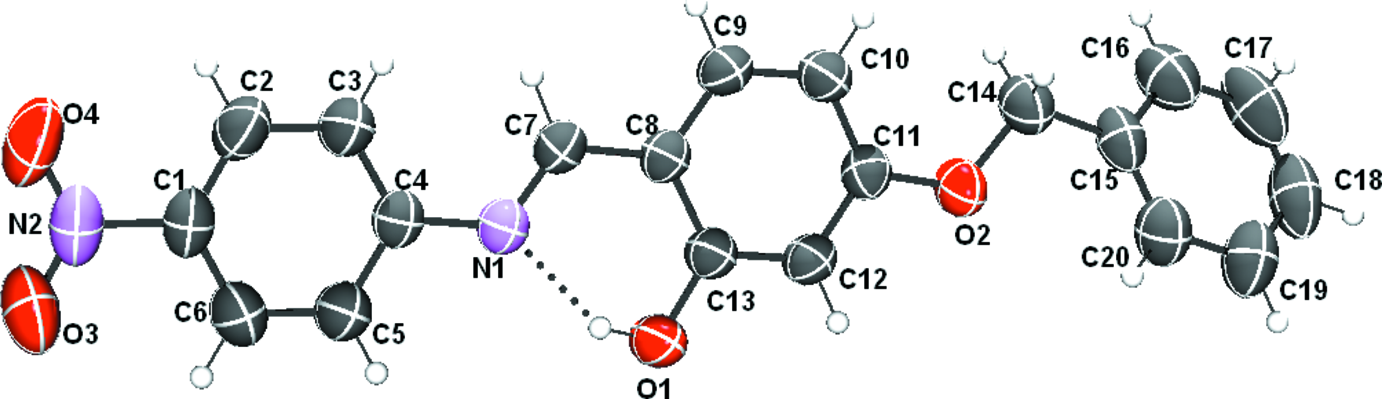

Supplement: Supplementary file 4 [file e-71-o1000-fig1.tif]

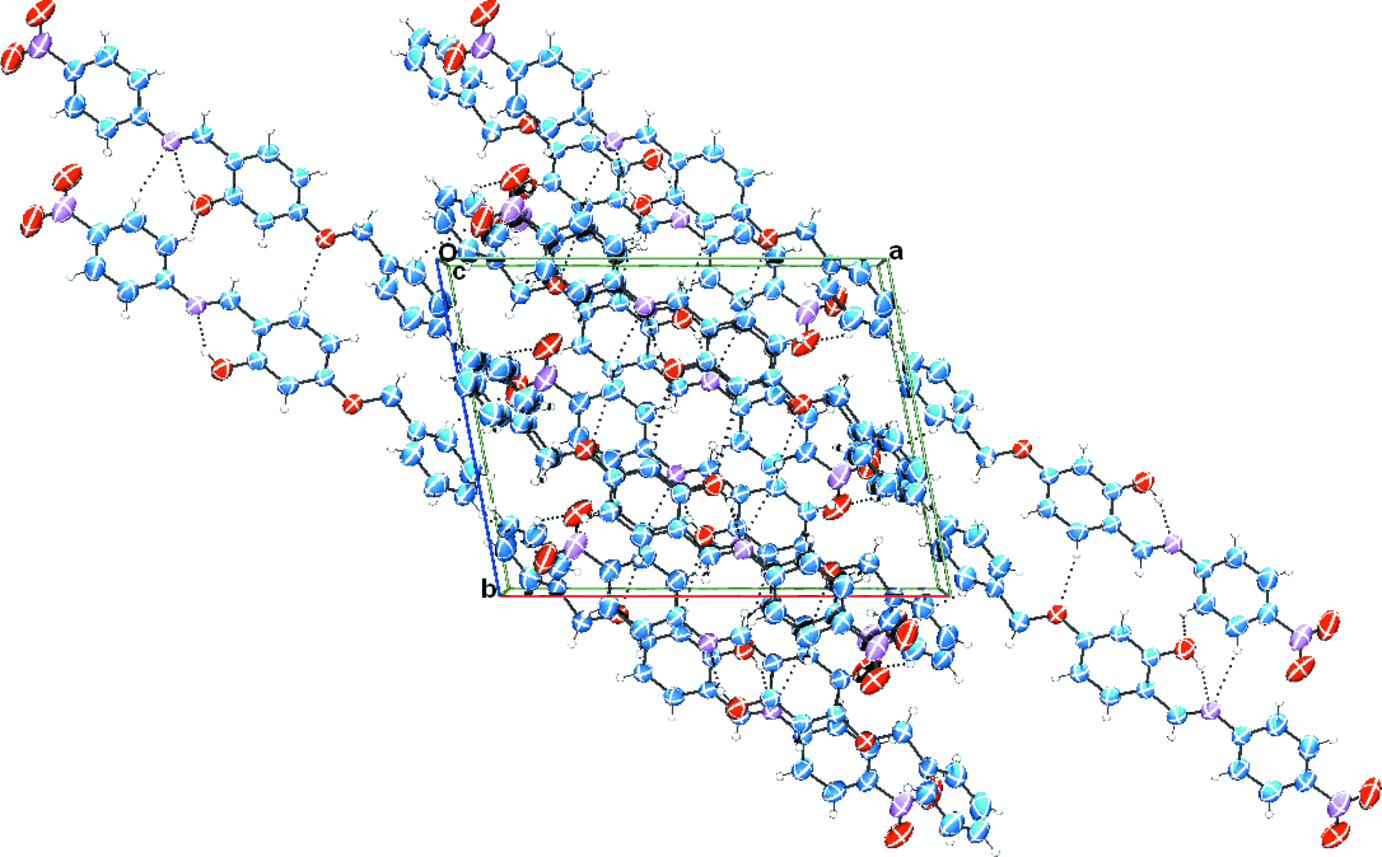

Supplement: Supplementary file 5 [file e-71-o1000-fig2.tif]
